# Targeted Next-Generation Sequencing of 117 Routine Clinical Samples Provides Further Insights into the Molecular Landscape of Uveal Melanoma

**DOI:** 10.3390/cancers12041039

**Published:** 2020-04-23

**Authors:** Sophie Thornton, Sarah E. Coupland, Lisa Olohan, Julie S. Sibbring, John G. Kenny, Christiane Hertz-Fowler, Xuan Liu, Sam Haldenby, Heinrich Heimann, Rumana Hussain, Natalie Kipling, Azzam Taktak, Helen Kalirai

**Affiliations:** 1 Liverpool Ocular Oncology Research Group, Department of Molecular and Clinical Cancer Medicine, Institute of Translational Medicine, University of Liverpool, Liverpool L7 8XT, UK; s.e.coupland@liv.ac.uk (S.E.C.); h.kalirai@liv.ac.uk (H.K.); 2 Liverpool Clinical Laboratories, Liverpool University Hospitals NHS Foundation Trust, Liverpool L69 3GA, UK; natalie.kipling@liverpool.ac.uk; 3 Centre for Genomic Research, Institute of Integrative Biology, University of Liverpool, Liverpool L69 7BT, UK; lolohan@gmail.com (L.O.); julie.sibbring@liverpool.ac.uk (J.S.S.); John.Kenny@teagasc.ie (J.G.K.); C.Hertz-Fowler@wellcome.ac.uk (C.H.-F.); xuanliu@liverpool.ac.uk (X.L.); S.Haldenby@liverpool.ac.uk (S.H.); 4 St. Paul’s Eye Unit, Liverpool University Hospitals NHS Foundation Trust, Liverpool L7 8XP, UK; hheimann@liverpool.ac.uk (H.H.); rumanahussain@hotmail.com (R.H.); 5 Department of Medical Physics and Clinical Engineering, Liverpool University Hospitals NHS Foundation Trust, 1st Floor Duncan Building, Liverpool L7 8XP, UK; afgt@liv.ac.uk

**Keywords:** next-generation sequencing, uveal melanoma, prognostication, mutation, clinical samples, chromosome, copy number

## Abstract

Uveal melanoma (UM) has well-characterised somatic copy number alterations (SCNA) in chromosomes 1, 3, 6 and 8, in addition to mutations in *GNAQ, GNA11, CYSLTR2, PLCB4, BAP1, SF3B1* and *EIF1AX*, most being linked to metastatic-risk. To gain further insight into the molecular landscape of UM, we designed a targeted next-generation sequencing (NGS) panel to detect SCNA and mutations in routine clinical UM samples. We compared hybrid-capture and amplicon-based target enrichment methods and tested a larger cohort of primary UM samples on the best performing panel. UM clinical samples processed either as fresh-frozen, formalin-fixed paraffin embedded (FFPE), small intraocular biopsies or following irradiation were successfully profiled using NGS, with hybrid capture outperforming the PCR-based enrichment methodology. We identified monosomy 3 (M3)-UM that were wild-type for *BAP1* but harbored *SF3B1* mutations, novel frameshift deletions in *SF3B1* and *EIF1AX*, as well as a *PLCB4* mutation outside of the hotspot on exon 20 coinciding with a *GNAQ* mutation in some UM. We observed samples that harboured mutations in both *BAP1* and *SF3B1*, and *SF3B1* and *EIF1AX*, respectively. Novel mutations were also identified in *TTC28, KTN1, CSMD1* and *TP53BP1*. NGS can simultaneously assess SCNA and mutation data in UM, in a reliable and reproducible way, irrespective of sample type or previous processing. *BAP1* and *SF3B1* mutations, in addition to 8q copy number, are of added importance when determining UM patient outcome.

## 1. Introduction

Uveal melanoma (UM), the most common primary intraocular malignancy in adults, has an incidence of 3–8 individuals per million per year in Caucasians [1,2]. Despite successful treatment of the primary tumor with surgery and/or radiotherapy, metastatic death occurs in ~50% of patients [3,4]. Stratifying UM patients on the basis of their metastatic-risk is essential for efficient, personalised care. In Liverpool, UM patients are currently stratified into metastatic-risk groups—i.e., low (LR) or high (HR) risk—using a combination of clinical, histopathological and genetic factors [5,6]. Patients with HR-UM undergo regular liver imaging, using magnetic resonance imaging (MRI) to enable the early detection of metastases, and thereby enhance opportunities for liver resection and enrolment into clinical trials [5]. Liver resection has been shown to prolong the median survival of UM patients by 19 months compared with patients treated palliatively [7]. Conversely, patients with LR-UM can be reassured and avoid long-term surveillance, and there are proven benefits both to them and to health service providers [3].

Distinct somatic copy number alterations (SCNA) occur in UM, the most common being monosomy 3 (M3) [8]. This corresponds with a significantly worse prognosis, especially when accompanied by polysomy chromosome (chr) 8q [9,10]. Increasing copies of chr 8q significantly correlate with reduced survival, in a dose-dependent fashion [11]. SCNA in chr 1p, 6p and 6q have also been linked with survival outcomes [11,12,13,14].

In addition to these well-characterised SCNA, UM has two sets of driver mutations: one which initiates tumorigenesis in the form of mutually exclusive gain-of-function mutations in *GNAQ*, *GNA11*, *CYSLTR2* or *PLCB4,* major players in the Gq signalling pathway [15,16,17,18]; and the other consists of mutations in *BAP1* [19], *SF3B1*/*SRSF2* [11,20] and *EIF1AX* [20], which have been correlated with high-, intermediate- and low-metastatic risk groups, respectively [11]. Inactivating mutations in *BAP1* are closely associated with HR-M3 UM, with recent data suggesting that the bi-allelic inactivation of *BAP1* is required to influence prognosis [21]. Missense mutations in splicing factor *SF3B1* are often observed in disomy 3 (D3) UM and have been shown to predispose patients to late-onset metastatic disease [22]. Similarly, mutations in *SRSF2*, another member of the spliceosome, are observed in D3-UM, suggesting there are some functional similarities between *SRSF2*- and *SF3B1*-mutant UM [11]. Mutations in *EIF1AX* are mainly observed in D3-UM and are associated with LR-UM [23]. Other mutations in *FBXW7* [20], *DLK2*, *CSMD1*, *KTN1*, *TP53BP1*, *TTC28* [14] and *MAPKAPK5* [11] have also been observed at low frequencies; however, their clinical significance remains unknown.

Recent genomic studies reported that UM could be subdivided into four main groups using unsupervised hierarchical clustering according to genetic alterations (SCNA, mutations and RNA-Seq), which were associated with an increasingly poor prognosis [11,14]. Based on these findings, there have been several efforts to design targeted next-generation sequencing (NGS) panels specifically for UM. In 2017, a bespoke NGS panel was designed to examine mutations in skin melanoma and UM simultaneously; however, this only examined mutations in *GNAQ* and *GNA11*, which are not associated with patient prognosis [24]. Another panel combined SCNA analysis of chromosomes 1, 3 and 8 and mutation analysis of *GNAQ*, *GNA11*, *BAP1*, *SF3B1* and *EIF1AX* using the Ion Torrent (Thermofisher Scientific) sequencing platform [25]. More recently, a pan-cancer sequencing panel consisting of 500 genes frequently mutated in cancer (including those frequently mutated in UM) was used to analyse 62 non-irradiated biopsies and fresh resection UM samples [26], and also in another study, 35 matched primary UM and their metastases [27]. The studies reported the successful detection of SCNA and mutations that may enhance survival prognostication. Castle Biosciences have also developed a 7-gene NGS panel ‘DecisionDx-UMSeq’, although to our knowledge this has not been tested with formalin-fixed paraffin embedded (FFPE) or irradiated material.

This study details the largest cohort of UM-patients to be analysed using a targeted NGS panel to date. We examined the ability of NGS to detect both SCNA in chr 1, 3, 6 and 8, and mutations in *GNAQ*, *GNA11*, *CYSLTR2*, *PLCB4*, *BAP1*, *SF3B1*, *SRSF2*, *EIF1AX*, *FBXW7*, *DLK2*, *CSMD1*, *KTN1*, *TP53BP1* and *TTC28*, in irradiated UM, as well as in FFPE tumor samples. Hybrid capture and PCR-based enrichment methods for NGS were initially compared. Following this, the best technology was chosen for the evaluation of a larger UM cohort, and all genetic data were correlated with clinical and histopathological features, and with patient outcome. 

## 2. Results

### 2.1. Patient and Tumor Demographics

**Figure 1:**
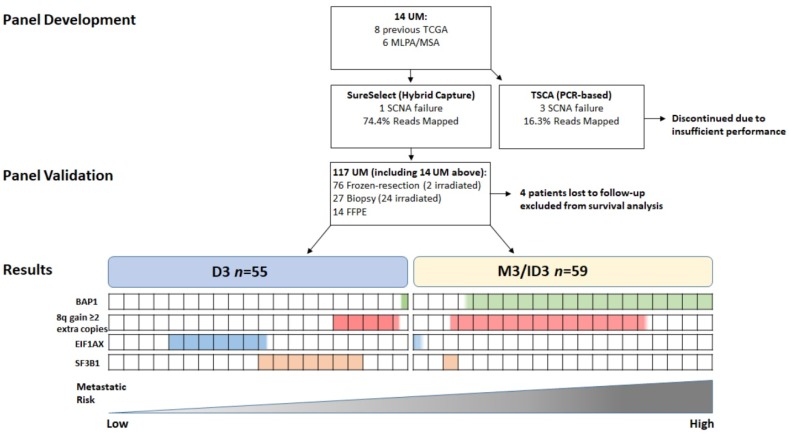
Flowchart of 117 UM specimens examined in the present study: *n* = 76 frozen-resection (2 post-irradiation); *n* = 27 Biopsy (24 post-irradiation); *n* = 14 FFPE. Four patients were lost to follow-up and excluded from survival analysis. *n* = 55 were D3, and *n* = 59 were M3 or ID3. Proportion of cases with the genetic alteration listed are highlighted by the coloured boxes. Each box represents 5% of UM patients examined.

DNA from primary UM samples with a median follow-up of 65 months (range 0–132 months) were from 117 consenting patients treated at the Liverpool Ocular Oncology Centre (LOOC), Liverpool University Hospitals NHS Foundation Trust. Of the UM samples analysed, 27/117 (23%) were biopsies that had residual DNA available (stored at −80 °C), 14/117 (12%) specimens were FFPE and 76/117 (65%) were frozen, resection samples from which DNA could be extracted. Twenty-six cases were selected, as they were taken post-irradiation with either ruthenium plaque radiotherapy (PRXT) or proton beam radiotherapy (PBR) (Figure 1). All samples had previously undergone routine genetic testing by either multiplex ligation dependent probe amplification (MLPA) or microsatellite analysis (MSA). 

The study consisted of 63 males and 54 females with a median age of 64; range 16–87 years (mean age 62 years) at the time of management of their primary UM. Primary management was enucleation in 78/117 (66%) UM patients; local resection 12/117 (10%); endoresection 1/117 (1%); PRXT 16/117 (14%); and PBR in 10/117 (9%). Secondary treatment was necessary for 4/117 (4%) UM patients, as a result of tumor recurrence (Table 1). Figure 1 describes the flow of patients through this study. 

The UM median largest basal diameter (LBD) was 15.0; range 4–22 mm (mean 14.6 mm) with a median ultrasound height (UH) 7.5; range 1–15.7 mm (mean 7.5 mm) (Table 1). The American Joint Committee on Cancer (AJCC) stage was: 14/117 (12%) stage 1, 26/117 (22%) stage 2, 57/117 (49%) stage 3 and 20/117 (17%) stage 4. Ciliary body involvement was reported in 36/117 (31%) cases and extraocular UM extension was present in 9/117 (8%) of cases. Epithelioid cells were seen in 50/117 (43%) of cases with the remaining 67/117 (57%) having a spindle cell morphology. Full histological assessment was only undertaken in resection specimens (enucleation or local resection samples; *n* = 90), which had a mean mitotic count of 7/40 hpf (median 5/40 hpf; range 1–72/hpf). Closed Periodic Acid Schiff (PAS) and connective tissue loops were identified in 47/90 (52%) cases, and focal necrosis was observed in 21/89 (24%) cases. At study closure (23 September 2019), 62/117 (53%) patients were alive without evidence of metastasis, 40/117 (34%) patients had died from metastatic disease, 11/117 (10%) patients died from other causes and 4/117 (3%) patients were lost to follow-up.

### 2.2. Panel Comparison (14 Samples)

Of the initial 14 UM samples analysed for panel comparison, 1/14 (7%) and 3/14 (21%) failed to produce reportable SCNA data with the SureSelect (SureSelect XT HS using SureDesign, Agilent) and TSCA (TruSeq Custom Amplicon using DesignStudio Illumina) panels, respectively. 13/14 (93%) UM samples had available SCNA data from previous MLPA for chr1, 3, 6 and 8; the remaining sample was tested by MSA for chr3 status only. There was 100% agreement for chr3 status between the MLPA/MSA data and that provided by both NGS tests in this initial sample cohort (Table S1—samples marked by an asterisk). There was 100% concordance for *GNAQ*, *GNA11*, *BAP1*, *SF3B1* and *EIF1AX* mutations between both testing platforms. No false positives were detected in any of the samples. Of note, 6/14 UM test samples had been previously submitted by our group to the TCGA-UM study, and there was also 100% concordance for all mutations identified. The SureSelect panel was chosen to test the larger UM cohort, due to its greater success rate in SCNA analysis and better coverage (Table S2). 

### 2.3. Mutation Frequency

In total, 117 UM samples (including the 14 initial samples analysed) were sequenced using the above bespoke SureSelect NGS panel. This included 26 UM that had previously undergone PBR or PRXT and for which mutation data was successfully obtained. Initiating mutations occurred in 62/117 (53%) for *GNAQ*; 42/117 (36%) for *GNA11*; 2/117 (2%) for *CYSLTR2* and 1/117 (1%) for *PLCB4*, which was concomitant with a *GNAQ* mutation (Table S3). Driver mutations occurred in 50/117 (43%) for *BAP1* (1/50 (2%) occurring in a D3-UM); 25/117 (21%) for *SF3B1* (3/25 (12%) coincided with a *BAP1* mutation 2/25 (8%) coincided with an *EIF1AX* mutation, 5/25 (20%) had partial loss or M3); 22/117 (19%) for *EIF1AX* (2/22 (9%) occurring in a M3-UM). Interestingly, two D3-UM were found to have concurrent *EIF1AX* and *SF3B1* mutations. 

Novel mutations were observed in: *PLCB4*: 1/117 p.Met549_Gly556delinsIle; *KTN1*: 2/117 p.Pro195Thr p.Gln86dup; *TTC28*: 4/117p. Arg21*, p.Pro1216His, p.Ala18Gly and p.lleI1296Val; *CCMD1*: 2/117 p.Pro1097His, p.Pro108Leu; *TP53BP1*: 2/117 p.Ile455_Pro456del and p.Glu1529*. These rare variants were confirmed using Integrative Genomics Viewer with a minimum allele frequency of 30%. No mutations were detected in any of the cases for the genes *BRAF, DLK2, FBXW7* or *SRSF2*. 

### 2.4. SCNA Analysis and Comparison with MLPA/MSA

We compared the SCNA datasets to establish whether the SureSelect NGS panel accurately detected SCNA in chr1, 3, 6 and 8 when analysed by MLPA and for chr3 when analysed by MSA. One sample failed to provide clear SCNA data by NGS and was excluded from the concordance data below, as were SCNA deemed ‘unclassifiable’ by MLPA. Concordance was observed with NGS as follows: chr1p—81/98 (83%); chr3—103/112 (92%); chr6p—68/88 (77%); chr6q—77/99 (78%); chr8p—64/102 (63%); and chr8q—72/97 (74%) (Table S1). 

SCNA data from the NGS panel was successfully obtained from both non-irradiated and irradiated samples and demonstrated: loss of 1p in 25/116 (22%) with 8/25 (32%) coinciding with a concomitant gain of 1q; gains in 1q in 9/116 (8%); M3 in 55/116 (47%); isodisomy 3 (ID3) in 2/116 (2%); loss of 3p in 1/116 (1%) and loss of 3q in 1/116 (1%), subsequently categorised as partial loss of chr3 (PL3); 6p gain in 46/116 (40%) cases with 37/46 (80%) occurring with D3 and 9/46 (20%) occurring with M3/ ID3/PL3; 6q loss in 25/116 (22%) of samples with 12/25(48%) occurring with M3/isodisomy 3/PL3; 8p loss in 20/116 (17%) each with a concomitant gain of 8q (Table S3). A complete gain of chr8 was seen in 36/116 (31%) UM. Gain of chr8q only occurred in 75/116 (65%) samples; 24/75 (32%) in D3 UM and 51/75 (68%) in M3/ID3/PL3 UM. 8q gain varied with respect to number of extra copies: the median was two extra copies for both M3/ID3/PL3 and D3 UM ranging from 1 to 9 in the former group and from 1 to 4 in D3 UM. 

### 2.5. Cox Regression

Univariate analysis was carried out using a significance level of *p* < 0.005 after Bonferroni correction.

Factors significantly associated with survival were: epithelioid cytomorphology, LBD, UH, ciliary body involvement, *BAP1* and chr3 status (Table 2). These variables were entered into the Cox model and backward selection of covariates was carried out using the likelihood ratio to determine ‘goodness of fit’ of the model. At the 0.01 significance level, chr3 loss was significantly associated with reduced survival (*p* ≤ 0.001) with a hazard ratio of 5.949 (Table 3). 

### 2.6. Survival

**Figure 2:**
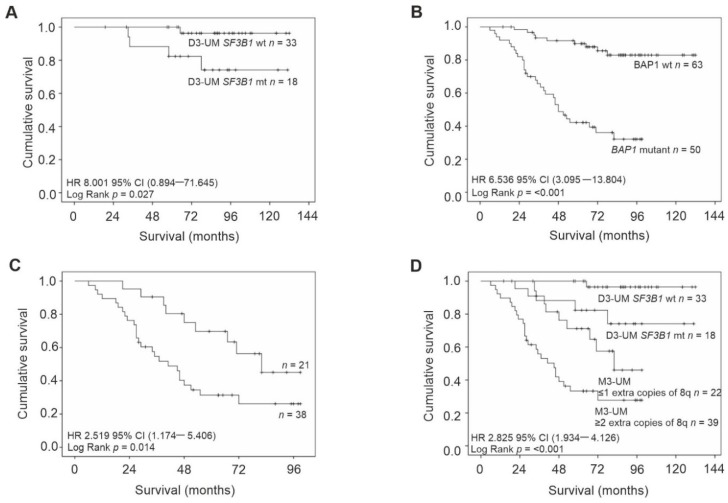
Kaplan–Meier survival curves estimate survival in UM patients stratified by: (**A**) *SF3B1* wild-type/mutation status in D3-UM *n* = 51 (Log Rank, *p* = 0.027); (**B**) *BAP1* wild-type/mutation status *n* = 113 (Log Rank, *p*  <  0.001); (**C**) extra copies of chr 8q in M3/ID3-UM *n* = 59 (Log Rank, *p* = 0.014) and (**D**) *SF3B1* wild-type/mutation status in D3-UM *n* = 51 and extra copies of chr 8q in M3/ID3-UM *n* = 59 (Log Rank, *p*  <  0.001). Number of events indicates the number of deaths due to metastatic melanoma. Log Rank tests were used to compare survival across groups.

Kaplan–Meier survival curves and tables were examined for all primary UM stratified according to: chr3 status, extra copies of chr8q, and mutations in *BAP1* and *SF3B1*. The following were significantly associated with a reduced survival time: loss of chr3 (Log Rank *p*  <  0.001), *BAP1* mutations (Log Rank *p*  <  0.001), M3-UM with more than two copies of 8q (Log Rank *p*  =  0.014) and D3-UM with *SF3B1* mutations (Log Rank *p*  =  0.027) (Figure 2). 

### 2.7. BAP1 IHC

Seventy of the ninety surgical UM samples (enucleation/local resection) had previously undergone routine immunohistochemistry (IHC) to determine nuclear BAP1 (nBAP1) protein expression; the remaining samples did not have enough material for subsequent IHC analysis. nBAP1 protein was absent in 38/70 cases (54%) of which 31 (82%) UM also had mutations in the *BAP1* gene. Of the 7/38 (18%) UM with no *BAP1* mutations, four patients had M3-UM and three had died from metastatic disease. Furthermore, 3/32 (9%) UM positively expressed nBAP1 protein but had clear mutations in *BAP1*, all of which were missense alterations (q.Glu31Lys, q.Cys91Gly and q.Ala142Pro). 

### 2.8. SF3B1 Mutations in M3 UM

*SF3B1* mutations have previously been associated with D3-UM with late onset metastasis [22]. In our cohort, 5/25 cases (20%) with *SF3B1* mutations died of metastatic UM at the time of study closure. Of these five cases, four tumors were D3-UM and one was a M3-UM with a *BAP1* mutation. To investigate the prevalence of *SF3B1* mutations in M3-UM that lacked mutations in *BAP1,* we identified 20 additional cases of M3-UM where DNA was available and previous IHC analysis had demonstrated strong nBAP1 positivity, correlating with wild-type *BAP1* [28]. This additional UM cohort consisted of 12 males and 8 females with a mean age of 62 years at primary management (median age 62; range 45–80 years). The mean follow-up period was 48 months (median 61 months; range 6–79 months). Primary management was enucleation 17/20 (85%) and local resection 3/20 (15%). The mean LBD was 14.8 mm (median LBD 14.7; range 9.8–22.7 mm) with a mean UH of 8.0 mm (median UH 8.4; range 1.7–12.4 mm). Full histological assessment is detailed in Table S4. Of these additional 20 UM, 5 (25%) had mutations in *SF3B1;* 3/5 (60%) q.Arg625Cys and 2/5 (40%) q.Arg625His. At study closure, all five patients were alive; of interest, one patient developed liver metastases 40 months after primary management but underwent metastasectomy and is still alive 25 months after surgery.

## 3. Discussion

This is the largest study to date to profile UM using bespoke targeted NGS panels. It identified chr3 as the most significant factor associated with metastatic death and demonstrated for the first time that irradiated UM samples can be successfully profiled using NGS with no observable differences in quality when compared to non-irradiated UM samples. We identified a subset of M3-UM-patients without nBAP1 loss that demonstrated mutations in *SF3B1* and also describe concurrent disruptive frameshift deletions in *SF3B1* and *EIF1AX*. This is consistent with the observation in one case sequenced in TCGA that harboured both an *EIF1AX* and an atypical *SF3B1* (T663P) mutation [11]. We also observed co-occurring mutations in *BAP1* and *SF3B1* and *EIF1AX* and *SF3B1*. Novel mutations were also identified in *TTC28*, *KTN1*, *CSMD1* and *TP53BP1*. Of interest, we identified a mutation in *PLCB4* that does not fall within the hotspot on exon 20 and coincides with a *GNAQ* mutation. Furthermore, chr3 results obtained using the NGS panel were comparable to previous MLPA and MSA analyses. We recommend that this bespoke NGS panel ultimately replaces MLPA/MSA testing in routine labs, with the possibility of incorporating molecular data into prognostic tools—e.g., the LUMPO (Liverpool Uveal Melanoma Prognosticator Online), which was recently externally validated in a multicentre study [29].

### 3.1. Enrichment Comparison

Hybrid capture and PCR-based enrichment methods in NGS vary in how targeted regions are enriched [30]. Hybrid capture methodologies like the SureSelect XT HS used in this study, involve shearing gDNA into smaller fragments, library preparation and hybridisation with targeted biotinylated RNA baits. Using magnetic streptavidin beads, these baits can be separated, and the hybridised library amplified; whilst PCR-based methods hybridise a custom oligo pool flanking-targeted regions on unfragmented gDNA. These are then extended and ligated, and PCR is performed to integrate indexes and sequencing primers. The PCR-based method has the advantages of requiring lower DNA inputs with shorter preparation times. In our study, hybrid capture outperformed the PCR-based enrichment in terms of a larger percentage of reads mapped and a greater mean depth of coverage. Although there were no differences in the ability to call single nucleotide variants (SNV), there was an increased SCNA analysis failure rate for the PCR-based method. Similar comparison investigations in other cancer types found limited sensitivity of PCR-based sequencing, with several variants being missed due to regions of high guanine-cytosine content and suboptimal PCR conditions, yielding a minimal coverage not found when using hybrid capture [31,32,33]. An increased incidence of false positives and missed variants in PCR-based enrichment was also reported when evaluating hybrid capture versus PCR-based methods for whole-exome sequencing [34]. In contrast to our comparison, neither study found differences between the success rates of SCNA analysis. 

### 3.2. Comparison with Previous MLPA

In the current study, we were able to successfully examine both SCNA and SNV using a single NGS assay in fresh, FFPE and also irradiated tissues. Only one sample failed to produce a clear genotype, but this was expected because of a low yield of library post-capture. Furthermore, 10/116 (9%) UM samples were discordant with the original MLPA/MSA analyses for chr3: 2 were isodisomy 3, which had been classified as D3 by MLPA due to its limitations in detecting acquired homozygosity; two were shown to have regions of deletion not identified in previous MLPA, most likely due to an increased number of probes covering chr3 on the NGS panel. Of the remaining six discordant samples, four had been classified as M3 by MLPA but as D3 by NGS; two of these cases had *SF3B1* mutations but all patients were alive at the study closure. Two had been classified as D3 by MLPA but M3 by NGS; one had a *BAP1* mutation and both patients had died from metastatic disease. For chr1, 6 and 8, the discordance between the MLPA and the NGS SCNA was greater at 17–26% of UM cases, which is likely a result of the low probe coverage for these chromosomes on the MLPA panel. Whilst the median 8q copy number was the same in D3-UM and M3-UM, the 8q copy number burden was generally higher in M3-UM. This was reflected by a reduced survival in M3-UM with an 8q copy number of 4 or more consistent with previous reports that 8q dosage is an important predictor of outcome in UM [11,35]. 

### 3.3. Irradiated Samples

This is the first study to examine irradiated UM samples using a NGS panel. No diminished quality or ability to genotype these tumors was observed amongst these samples. This is consistent with our findings using MSA/MLPA to genotype irradiated UM [36,37,38]. 

### 3.4. BAP1 Mutations

The frequency of *BAP1* mutations in the present study was 43% in total, occurring in 82% of M3-UM; these data are consistent with the findings of others [11,14,19,25]. The presence of a *BAP1* mutation in UM was associated with a worse survival. We have previously reported that nBAP1^+^ M3-UM have a better prognosis as compared with nBAP1^−^ M3-UM [21]; however, interestingly in this current study, M3-UM that were wild-type for *BAP1* (10/57; 18%) did not correlate with an increased survival time as compared with M3-UM with *BAP1* mutations. This may be due to either the observation that *BAP1* mutations do not always correlate with loss of nBAP1 protein expression, or to the smaller cohort of patients in the present study [28,39]. 

### 3.5. SF3B1 Mutations

The frequency of *SF3B1* mutations in UM ranges in the literature from 11–34% [14,25], and in this study *SF3B1* mutations occurred in 21% of cases. *SF3B1* mutations are reported to occur mainly in D3-UM associated with late onset metastasis and decreased survival (22). This is consistent with our study in which 20/25 (80%) *SF3B1* mutations occurred in D3-UM with a significantly reduced survival time as compared with D3/*SF3B1*wt UM (*p* = 0.027). 

A novel disruptive frameshift deletion in *SF3B1* of 15 nucleotides was observed in p.Lys653_Ser657del on heat domain 4, outside the hotspot region of codon 625; the significance of this is unclear. Of particular interest in our study are five M3-UM or UM with PL of chromosome 3 with *SF3B1* mutations. Two of these UM harboured *BAP1* mutations, previously described in one other study (11); one patient succumbed to metastatic disease 12 months after primary management, and the second patient died of other causes 99 months (8.25 years) later. Three *SF3B1* mutations were recorded in M3-*BAP1wt* UM, a phenomenon only observed in one other study to date [11]. To examine this further, we tested an additional 20 cases of M3-UM with nBAP1 positivity and identified five cases with *SF3B1* mutations; at the time of study closure, all five patients were alive. Additional cases and longer follow-up are required to fully understand the clinical relevance of *SF3B1* mutations in M3-UM.

### 3.6. EIF1AX Mutations

*EIF1AX* mutations were detected in the present study in 19% of UM, which is consistent with that reported by other groups [11,14,18,25]. Interestingly, two UM demonstrated mutations in both *EIF1AX* and *SF3B1* despite previous reports describing that these occur in a mutually exclusive manner [11,25]. Of note, both patients died from metastatic disease at 34 and 58 months, respectively, after primary treatment. *EIF1AX* mutations are typically associated with D3-UM; however, we identified two M3-UM that displayed mutations in this gene. A novel disruptive frameshift deletion of 6 nucleotides from the coding sequence was also identified in p.Arg14_Gly15del of *EIF1AX*. 

### 3.7. Initiating Mutations

Mutations in *GNAQ* and *GNA11* occurred in 89% of UM in a mutually exclusive manner (53% and 39%, respectively), consistent with the literature [11,14,25]. Mutations predominantly occurred in exon 5 for *GNAQ* and *GNA11*, and two UM had mutations in exon 4. One sample contained two unusual mutations in exon 4 of *GNA11* p.R214K and p.R214S. These regions do not lie within any of the known functional domains of *GNA11* and have not been previously described; their effect on GNA11 protein function is unknown. Mutations in *CYSLTR2* were found in two UM in the hot spot region p.L129Q in exon 1 and occurred in a mutually exclusive manner to mutations in *GNAQ* and *GNA11,* as previously reported [17]. Consistent with our general understanding of the function of these mutations, there were no differences in survival outcome based on the mutational status of the driver mutations *GNAQ*, *GNA11* and *CYSLTR2*. 

Disruptive frameshift deletions in p.M549_G556delinsI and M561_G568delinsI mutations were observed in *PLCB4* in a single UM sample. These cases also showed a p.R183Q mutation in *GNAQ*. Previous studies identified recurrent mutations in *PLCB4* in a hot-spot region p.D630Y and p.D630N on exon 20 [18]. The mutation identified in our study occurred in exon 18 and is the first mutation in this region to be described in UM. Though it was initially thought that *PLCB4* mutations occurred in a mutually exclusive manner to *GNAQ*, *GNA11* and *CYSLTR2*, our study and that of Robertson et al. [11] demonstrate *PLCB4* mutations concurrent to *GNAQ* and *GNA11* mutations. 

### 3.8. Other Mutations

We observed low frequency (3%) somatic mutations in genes originally identified by Royer-Bertrand et al. (6%), namely in *TTC28*, *CSMD1*, *KTN1* and *TP53BP1* [14]. Most of these genes are involved in various cellular processes, e.g., cell cycle regulation [40], cell migration and proliferation [41,42], kinesin binding [43] and DNA double-strand break repair [44]. Our NGS panel was custom-designed to have full coverage of the *TTC28*, *CSMD1*, *KTN1* and *TP53BP1* genes, and because of its targeted nature had greater coverage in comparison to whole-exome sequencing methodologies. Due to their low frequency in this study, no association could be made between the mutations in *TTC28*, *CSMD1*, *KTN1* or *TP53BP1* and UM with particular clinical or morphological features. It is worth noting that previously described mutations in *SRSF2*, *DLK2* or *FBXW7* were not detected in this large study [14,20,45].

## 4. Materials and Methods

### 4.1. Patients

In this retrospective cohort study, primary UM samples were collected from 117 patients who were treated at the Liverpool Ocular Oncology Centre (LOOC), Liverpool University Hospitals NHS Foundation Trust, between January 2008 and May 2015. This time period was chosen to allow sufficient follow-up (median, 65 months). The follow-up period was calculated from the date of primary management to either study end (23 September 2019) or to death from metastatic disease or other causes. Patients were treated either by radiotherapy or surgical resection, and their UM was genotyped using either MLPA or MSA, as described below. 

### 4.2. Specimen Characteristics

Specimens consisted of DNA (stored at −80 °C) previously extracted from fresh biopsies all preserved in CytoLyt (Cytyc Corp) and stored at 4 °C, fresh-tumor tissue snap-frozen in liquid nitrogen and stored at −80 °C, and FFPE UM samples stored at room temperature. Twenty-six of the DNA samples analysed were post-irradiation specimens.

### 4.3. Study Design

The clinical endpoint examined in this study was death from metastatic disease. Patients who died from causes other than those relating to UM were included in the study, and data for these records were treated as right-censored cases for evaluation purposes. This study conformed to the principles of the Declaration of Helsinki and Good Clinical Practice guidelines. Approval for the study was obtained from the Health Research Authority South Central - Hampshire B Research Ethics Committee (REC ref 15/SC/0611). All samples and data were provided by the Ocular Oncology Biobank (REC ref 16/NW/0380). All patients had provided informed consent for the use of their samples and data in research.

### 4.4. Assay Methods

#### 4.4.1. Morphological/Histological Studies

All samples underwent routine histopathological and cytological workup assessing cell type, mitotic count, and presence of PAS+ connective tissue loops where possible (28). Furthermore, 90/117 enucleation and local resection specimens had a full histological workup, whilst 27/117 biopsies and endoresection specimens underwent cytological examination only. Additionally, IHC analysis of nBAP1 expression was undertaken in 70/117 cases, as described previously [21]. 

#### 4.4.2. DNA Extraction and Quantification

Methods for DNA extraction from FFPE and frozen UM have been published elsewhere [46]. DNA integrity of FFPE samples was qualified by performing a qPCR using the Agilent NGS FFPE QC Kit. (Agilent Technologies Inc., UK).

#### 4.4.3. Chromosomal SCNA Analysis

MLPA (MRC Holland, The Netherlands) and MSA were used to assess SCNA, and subsequent comparison with NGS data were undertaken during routine genetic testing of patient samples, as previously described [47,48]. Cases yielding >100 ng of DNA were tested using MLPA, whilst MSA was undertaken for UM samples with lower DNA yields. 

#### 4.4.4. Next-Generation Sequencing

Two custom NGS panels were designed: SureSelect XT HS using SureDesign (Agilent) and TruSeq Custom Amplicon (TSCA) using DesignStudio (Illumina). Both panels were designed to cover mutations in *GNAQ* (exons 4 & 5), *GNA11* (exons 4 & 5), *SF3B1* (exons 12 & 14), *EIF1AX* (exons 1 and 2), and all exons of *BAP1*, *FBXW7*, *DLK2*, *CSMD1*, *CYSLTR2*, *KTN1*, *TP53BP1*, *SRSF2*, *PLCB4*, *TTC28* and *BRAF* (negative control). Both enrichment methods included the incorporation of unique molecular identifiers or barcodes to reduce errors and quantitative bias introduced by the amplification process. For the SureSelect XT HS, additional probes were included to examine SCNA in chr1: 1541 probes; chr3: 1287 probes; chr6: 1094 probes; chr8: 933. The TSCA panel included additional probes to examine SCNA in chr3: 83 amplicons; chr6: 76 amplicons and chr8: 67 amplicons. Chr1 was not included in the TSCA NGS panel due to tiling limitations. As the panels were worked up on larger resection samples, the DNA input was 50 ng for both panels. Libraries were constructed using either the SureSelect XT HS Reagent and Capture Library Kit (Agilent Technologies Inc., United Kingdom) or TruSeq Custom Amplicon Low Input Kit (Illumina Inc., United Kingdom), according to the manufacturer’s instructions. The two panels were tested and compared using 14 frozen UM samples, 8 of which had been previously profiled by The Cancer Genome Atlas (TCGA) UM study [11], and 6 had available data from previous genotyping plus an additional two reference samples (Genome In A Bottle, HDx). 

The SureSelect XT HS was subsequently selected to test a larger cohort of 95 fresh and 13 FFPE UM samples with reference samples included in each sequencing run. The DNA input varied (5 ng–25 ng) depending upon the sample type.

#### 4.4.5. Sanger Sequencing

Exon 14 of *SF3B1* was sequenced using PCR-based capillary Sanger sequencing in an additional twenty M3-UM with unusual nBAP1+ protein expression [21]. Oligonucleotides were constructed by Eurofins Genomics; forward 5’-GGCCGAGAGATCATTTCT-3, reverse 5’-AAGAAGGGCAATAAAGAAGGA-3’, product size 289bp. PCR was performed in a reaction volume of 50 μL containing 100 ng of genomic DNA, 0.25 μL of Thermo-Start Taq DNA Polymerase (Thermo Scientific), 5 μL of HP Buffer, 4 μL of 25 mM MgCl_2_, 2 μL of dNTP (2 mM each), 31.25 μL Nuclease Free water and 1 μL of each of the primers. The thermal cycling profile was as follows: initial denaturation at 95 °C for 15 min and 35 rounds of amplification at 95 °C for 15 s, 55 °C for 30 s and 72 °C for 1 min. A final extension step at 72 °C for 5 min was added. PCR products were purified using the QIAquick PCR purification kit (Qiagen, United Kingdom) according to the manufacturer’s protocol. Sequencing of PCR products was carried out by GATC at Eurofins Genomics in accordance with ISO 17025. Sequencing data were analysed using Chromas Lite (2.1.1., Technelysium Pty Ltd.). 

### 4.5. NGS Data Analysis

NGS libraries were sequenced on the Illumina MiSeq platform (2 × 250 bp paired-end) by the Centre for Genomic Research (www.cgr.liv.ac.uk), University of Liverpool, UK. Base-calling and de-multiplexing of indexed reads were performed by CASAVA version 1.8.2 (Illumina) to produce the raw sequence data in FASTQ format. The raw FASTQ reads were trimmed to remove Illumina adapter sequences using Cutadapt version 1.2, and low-quality bases using Sickle version 1.200.

Trimmed reads were aligned to the human GRCh37 reference genome (ftp://ftp-trace.ncbi.nih.gov/1000genomes/ftp/technical/reference/phase2_reference_assembly_sequence/hs37d5.fa.gz) with the short-read alignment tool, BWA-MEM (version 0.7.5a-r405). Following alignment, PCR and optical duplicate reads were identified and removed with UMI-tools (https://github.com/CGATOxford/UMI-tools). Subsequently, the Genome Analysis Toolkit (GATK) (version 3.7) Indel Re-Aligner module was used to locally realign reads around the putative insertion and deletion sites. GATK BaseRecalibrator module was used for recalibrating the base calls. The aligned data were then analysed using tCoNut (https://github.com/tgen/tCoNuT) to detect SCNAs. The variants were called by GATK and annotated by SNPeff.

### 4.6. Statistical Analysis Methods

Survival time (months) was calculated from the date of primary management until death from metastases or study closure on 23 September 2019. Median survival time was estimated using the Kaplan–Meier product limit method. Univariate associations between survival time, clinical, histological and genetic features were examined using Cox proportional hazards regression models. Analyses were undertaken using SPSS Statistics v.24 (IBM), Microsoft R 3.5.1 and the packages rms, cmprsk and mstate. Cut-offs for SCNA used established values based on previous clustering analysis carried out at our centre: log rank < 0.85 loss, > 1.15 amplification [49]. The allelic frequency threshold to call a mutation was 10%.

## 5. Conclusions

Our bespoke UM NGS panel enables detailed SCNA and mutational information to be obtained from small UM biopsies, FFPE material and previously irradiated UM. This is in distinct contrast to some current methodologies, which, when applied to biopsies, can only determine chr3 status due to the low DNA yield. Moreover, consistent with other reports, *BAP1* and *SF3B1* mutations in addition to 8q copy number are of added importance when determining patient outcome and moves UM stratification away from a binary genetic classification based on chr3 copy number only. Identifying metastatic risk groups with greater precision than is currently possible with SCNA assessment alone will have implications on the frequency at which patients are followed up for subsequent liver imaging, and the imaging techniques applied, as well as on patient selection for clinical trials. Although at present, mutations in UM are not therapeutically actionable, it is hoped that continued advances in our understanding of this disease will result in the use of these biomarkers to predict response to emerging therapies. 

## Figures and Tables

**Table 1:** Patient and tumor demographics of *n* = 117 UM patients treated at Liverpool Ocular Oncology Centre.

Variable	Value (% or Range)
Age at PM (years)	
Median	64 (16–87)
Gender	
Female	54 (47%)
Male	63 (53%)
Survival	
Alive	62 (53%)
Death from MUM	40 (34%)
Death other causes	11 (10%)
Lost to follow-up	4 (3%)
Median (months)	65 (0–132)
Largest basal diameter (mm)	
Median	15.0 (4–22)
Ultrasound height (mm)	
Median	7.5 (1–15.7)
Ciliary body involvement	
Yes	36 (31%)
No	81 (69%)
Extra-ocular extension	
Yes	9 (8%)
No	108 (92%)
Epithelioid cells	
Yes	50 (43%)
No	67 (57%)
Closed loops present	
Yes	47 (40%)
No	43 (37%)
Not assessed	27 (23%)
Necrosis	
Yes	21 (17%)
No	68 (59%)
Not assessed	28 (24%)
Mitotic count per 40 high power field	
Median	5 (1–72)
Primary Management	
Enucleation	78/117 (66%)
Local Resection	12/117 (10%)
Endoresection	1/117 (1%)
Proton Beam RXT	10/117 (9%)
Ruthenium Plaque RXT	16/117 (14%)

**Table 2:** Univariate analysis of *n* = 117 UM patients treated at Liverpool Ocular Oncology Centre.

Variable	Sig.	Hazard Ratio (HR)	95.0% CI for HR	
			Lower	Upper
Age at PM	0.605	1.006	0.983	1.031
LBD	≤0.001	1.229	1.107	1.365
UH	≤0.001	1.198	1.086	1.322
CBI	0.003	2.602	1.396	4.849
EOE	0.183	2.024	0.717	5.715
Epithelioid	0.001	4.552	1.910	10.850
Chr 3	≤0.001	9.236	3.602	23.683
Extra copies 8	0.018	2.519	1.174	5.406
*SF3B1*	0.131	0.486	0.190	1.241
*BAP1*	≤0.001	6.536	3.095	13.804
*EIF1AX*	0.029	0.269	0.830	0.875

**Table 3:** Multivariate analysis of *n* = 117 UM patients treated at Liverpool Ocular Oncology Centre.

Variable	Sig.	Hazard Ratio (HR)	95.0% CI for HR	
			Lower	Upper
UH	0.016	1.124	1.022	1.235
Chr3	≤0.001	5.949	2.226	15.898
Epithelioid	0.059	2.375	0.969	5.825

## Supplementary Materials

The following are available online at https://www.mdpi.com/2072-6694/12/4/1039/s1, Table S1: Concordance Data, Table S2: NGS Quality Comparison, Table S3: Clinical, Molecular and Histopathological data 117 patients, Table S4: Monosomy 3 SF3B1 Cohort Data.
