# Supplementary material for: Targeted Next-Generation Sequencing of 117 Routine Clinical Samples Provides Further Insights into the Molecular Landscape of Uveal Melanoma
Source: Cancers (Basel). 2020 Apr 23;12(4):1039. doi: 10.3390/cancers12041039 (PMC7226611; doi:10.3390/cancers12041039)
Supplement: Supplementary file 1 [file cancers-12-01039-s001.zip › cancers-744518- supplementary Tables/cancers-744518-Table S3.pdf]

Supplementary Materials: Targeted Next-Generation Sequencing of 117 Routine Clinical Samples Provides Further Insights into the Molecular Landscape of Uveal Melanoma.

Sophie Thornton, Sarah E. Coupland, Lisa Olohan, Julie S. Sibbring, John G. Kenny, Christiane Hertz-Fowler, Xuan Lui, Sam Haldenby, Heinrich Heimann, Rumana Hussain, Natalie Kipling, Azzam Taktak and Helen Kalirai1

Table S1. Concordance Data

Table S2. NGS Quality Comparison

Table S3. Clinical, Molecular and Histopathological data 117 patients

Enrichment Method	Reads Mapped %	Reads Mapped % after UMI Removed	Number of Bases with Coverage More Than 0 (%)	Mean Depth of Coverage for the Bases with Coverage More Than 0
PCR	92.06	16.33	92.99	302x
Hybrid Capture	97.68	74.37	99.99	497.25x

Table S4. Monosomy 3 SF3B1 Cohort Data.

© 2020 by the authors. Submitted for possible open access publication under the terms and conditions of the Creative Commons Attribution (CC BY) license (http://creativecommons.org/licenses/by/4.0/).
